# Tuberculosis preventive therapy in postpartum women with HIV modifies *M. tuberculosis*-specific and nonspecific immune responses

**DOI:** 10.3389/fimmu.2026.1722621

**Published:** 2026-03-11

**Authors:** Damayanti Yengkhom, Grace Montepiedra, Gaerolwe Masheto, Deo Wabwire, Lynda Stranix-Chibanda, Allen Matubu, Avy Violari, Gerhard Theron, Amita Gupta, Adriana Weinberg

**Affiliations:** 1 Department of Pediatrics, Aurora, CO, United States; 2 Department of Biostatistics, Harvard T.H. Chan School of Public Health (ret), Boston, MA, United States; 3 Botswana Harvard AIDS Institute Partnership, Gaborone, Botswana and Harvard T.H. Chan School of Public Health, Boston, MA, United States; 4 Makerere University-Johns Hopkins University Research Collaboration, Kampala, Uganda; 5 Faculty of Medicine and Health Sciences, University of Zimbabwe Clinical Trials Research Centre, Harare, Zimbabwe; 6 Perinatal HIV Research Unit, University of Witwatersrand, Johannesburg, South Africa; 7 Department of Obstetrics and Gynecology, Faculty of Medicine and Health Sciences, Stellenbosch University, Cape Town, South Africa; 8 Department of Medicine, Johns Hopkins University School of Medicine, Baltimore, MD, United States; 9 Departments of Medicine and Pathology, University of Colorado Anschutz Medical Campus, Aurora, CO, United States

**Keywords:** granzyme B, HIV, immunologic memory, innate cell immunologic memory, interferon γ, isoniazid, pregnancy, preventive therapy

## Abstract

**Background:**

Tuberculosis (TB) has significant morbidity in pregnant and postpartum women with HIV (pregnant and PPWWHIV). TB preventive therapy (TPT) is recommended in pregnant and PPWWHIV with documented or presumed latent TB infection (LTBI). TB-stimulated IFNγ release assay and skin test positivity decline after TPT, but the underlying mechanisms and relationship with TB-specific immunologic memory are incompletely understood. We investigated this aspect in PPWWHIV.

**Methods:**

PPWWHIV with LTBI received isoniazid TPT between 12 and 40 weeks postpartum. Blood obtained at 12 and 44 weeks postpartum was used to compare functional and phenotypic characteristics of unstimulated and TB-stimulated CD4+ and CD8+ conventional T cells (Tconv); unconventional T cells, including γδ, iNKT, MR1+ and MR1- MAIT, and NKT; NK; and antigen presenting cells (APC) pre- and post-TPT.

**Results:**

In 45 participants with medians of 477 CD4+ T cells/µL and <50 HIV RNA copies/mL of plasma on antiretroviral therapy, both Tconv and innate immune cells responded to TB antigenic stimulation *in vitro* with an increase in functional markers. TPT was associated with a pronounced decrease in the proportions of granzyme B-expressing Tconv and unconventional T cell subsets both in TB-stimulated and unstimulated conditions. TB-stimulated Th1- and Th17-like responses in unconventional T cells also decreased from pre- to post-TPT. TB-stimulated conventional and unconventional regulatory T cells mostly decreased after TPT with a few exceptions. Very few changes were observed in circulating or TB-stimulated APC in response to TPT.

**Conclusions:**

TPT was associated with a significant decrease in TB-specific T cell responses, including Tconv but mostly unconventional T cells, suggesting an important role of unconventional T cell memory in the control of TB infection. The prominent decrease of granzyme B-expressing T cells in response to TPT highlighted the importance of granzyme B in the maintenance of LTBI.

## Introduction

Tuberculosis disease (TB) is the leading cause of morbidity and mortality in people with HIV (PWHIV) (1, 2). Among cases of TB disease reported in 2023, 6.1% were in PWHIV. Moreover, among people who die of TB, the number of PWHIV is disproportionately high at 13% (3). Among PWHIV who are treated for TB, the rate of relapse is much higher than in people without HIV consistent with lower residual post-treatment protective immunity in PWHIV (4, 5). This finding suggests that PWHIV may lose their TB-specific memory immune responses after treatment.

Cell-mediated adaptive Immunity, particularly Th1 and Th17, have traditionally been considered the mainstay of protective immunity against TB (6, 7). These responses seem to be preferentially targeted for extinction by HIV infection (8). In addition, Th1 and Th17 effector T cells (Teff) require persistent stimulation provided by sustained antigenic exposure, which is generally eliminated with treatment of TB (9–13). These factors may contribute to the high relapse rate of TB in PWHIV. Due to the high morbidity and mortality of TB in PWHIV, the WHO recommends universal TB preventive therapy (TPT) for PWHIV in areas of high TB endemicity and for those with documented latent TB infection (LTBI) in areas of low endemicity. TPT was also shown to decrease the reactivity of TB skin tests (TST) and interferon γ (IFNγ) release assays (IGRA), suggesting decreases in TB-specific memory immune responses post-TPT (14–16). However, information in PWHIV is insufficient.

Recent studies have shown that innate immune cell memory responses may contribute to the immune protection against TB (17–24). Monocytes, macrophages, natural killer cells (NK), innate NKT cells (iNKT) and mucosal-associated innate T cells (MAIT) may acquire immunologic memory after exposure to TB or through BCG vaccination, which becomes instrumental to protection against TB (25). Little is known about the effect of TPT on innate cell immunologic memory or about TB-specific innate immunologic memory in PWHIV.

TB-specific immune responses in pregnant and postpartum women with HIV have also been insufficiently studied. Both pregnancy and postpartum are periods of high vulnerability to TB with a 2-fold higher incidence of TB disease compared to nonpregnant adults (26–28). Pregnancy is associated with increased immune regulation, which we and others have shown to decrease the reliability of IGRA for the diagnosis of TB infection in pregnancy (16, 29, 30). The potential amplification of the HIV- and pregnancy-associated decreases in Th1 responses against TB by TPT warrants further investigation in pregnant women with HIV.

To address the knowledge gaps enumerated above, we designed a study to test the hypothesis that TPT decreases TB-specific Th1 responses in postpartum women with HIV (PPWWHIV) and to investigate innate immunologic memory in this population. We obtained stored samples from participants enrolled in the TB APPRISE study, who received TPT between 12 and 40 weeks postpartum (31) and conducted a comprehensive analysis of TB-specific adaptive and innate immune cell responses in peripheral blood mononuclear cells (PBMC) before and after TPT.

## Participants and methods

### Study participants and samples

We used viably cryopreserved PBMC obtained from participants in TB APPRISE (NCT01494038) (31), which randomized PPWWHIV enrolled in the 2^nd^ or 3^rd^ trimester of pregnancy in a placebo-controlled double-blinded design to receive isoniazid TPT for 28 weeks starting at enrollment or starting at 12 weeks postpartum. Study participants were generally healthy albeit living with HIV. Women who initiated treatment during pregnancy received placebo between TPT completion and end of study. Women who initiated TPT postpartum received placebo from enrollment to 12 weeks postpartum. All women initiated antiretroviral therapy before or at study entry and continued through the entire study as per standard of care. The study received local ethics committee approval (individual clinical site information listed in the acknowledgments), and all women signed informed consent. At study entry, women had CD4+ T cell numbers, HIV plasma RNA, liver and renal function, and IGRA measured. Subsequently, CD4+ T cell counts, liver and renal function tests were repeated at monthly study visits. Blood was collected at study entry, 12 and 44 weeks post-partum for immunologic studies. The current study used cryopreserved PBMC obtained at 12 and 44 weeks postpartum from participants in the deferred treatment arm (started TPT at 12 weeks postpartum) who tested positive by IGRA at study entry.

### High dimensional flow cytometry

Adaptive and innate immune responses were examined using two 16-color flow cytometry panels. Cryopreserved PBMC were thawed as previously described (32). Cells with viability ≥70% were resuspended at 10^6^ PBMC/ml in RPMI 1640 (Corning) supplemented with 10% human AB serum (Gemini), HEPES buffer (Corning), 2mM L-glutamine (Gemini) and 1% penicillin and streptomycin (Gemini). Cells were incubated overnight at 37°C and 5% CO_2_ in a humidified atmosphere with 20 µg/ml *M. tuberculosis* cell membrane fraction (NR-14832 of strain CDC1551; BEI Resources, NIH) (33) or medium control. Brefeldin A (Sigma-Aldrich) was added for the last 4 h of incubation after which cells were washed, stained for viability and surface markers, fixed and permeabilized, and stained for intracellular markers with washes between each step. **Supplementary Tables 1**, **2** list the lineage and functional markers that were used to characterize each cell subset and the mAb used for each analyte, respectively. 200,000 cells were acquired using the Novocyte Quanteon (Agilent Technologies, Inc.) and analyzed with FlowJo 10.1. **Supplementary Figure 1** shows the gating strategies for T cells and APC. Each run included two sets of lab control PBMC with known performance characteristics to ensure inter-run reproducibility.

### Cytokine analysis in cell culture supernatants

PBMC culture supernatants were analyzed using MSD^®^ (Meso Scale Discovery) U-plex assays (K151AEM-1, K151XWK-1, and K15227N-1) according to the manufacturer’s instructions with modifications. Samples were diluted 2-fold for GM-CSF, CTLA-4, IL-10, IL-2, IFN-γ, IL-4, IL-17A, and PD1 analyses, and 250-fold for IL6 and TNFα using assay diluent. The TGF-β assay required acid treatment to activate TGF-β in the test samples, which was performed using 1 M HCl and 1.2 M NaOH in 0.5 M HEPES, resulting in a final dilution of 2.68-fold. Each run included supernatants obtained from lab control stimulated PBMC with known performance characteristics to monitor inter-run reproducibility.

### Statistical analysis

Comparisons of the frequencies of unstimulated and TB-stimulated cell subsets were performed using Friedman test with Benjamini, Krieger and Yekutieli false discovery rate (FDR) correction for multiple comparisons (34) and significance defined by FDR p<0.1. Additional analyses of TB-stimulated results after subtraction of unstimulated controls were performed using Wilcoxon matched-pairs rank signed test and significance defined by raw p<0.05. All analyses were performed in Prism version 10.1 (GraphPad).

## Results

### Demographic and HIV infection characteristics of the study population

Forty-five participants with paired samples available from pre- and post-TPT contributed specimens to this study. At entry, they had median age of 29 years (IQR of 24-34), median CD4+ cells/µL of 477, including 95% with >200 CD4+ T cells/µL, and 69% with undetectable plasma HIV RNA (**Table 1**). All participants were on 3-drug antiretroviral therapy at entry and continued through postpartum. Adherence, assessed by self-report, was >90%. Although data on HIV plasma RNA were not available after study entry, the CD4+ T cells increased over time, such that the average at 12 weeks postpartum was 52.6% and at 44 weeks postpartum 53.6%. The average CD4:CD8 ratio was 1.11 at 12 weeks postpartum and 1.15 at 44 weeks. All women who contributed samples to this study had positive IGRA results at entry as per study design.

**Table 1:** Characteristics of participants at study entry.

Characteristics	Median (quartiles) or N (%)
	N=45
Years of age	29 (24-34)
Country	
Botswana	8 (17.78%)
South Africa	37 (82.22%)
Weeks of gestational age at entry	27 (21-30)
14 to <24	17 (37.78%)
24 to ≤34	28 (62.22%)
Mid upper arm circumference (cm)	28 (26-30.7)
CD4+ cells/mm³	477 (379-594)
Participants with >200 CD4+ cells/mm³	43 (95.56%)
Participants with <50 HIV RNA copies/ml of plasma	31 (68.89%)
Antiretroviral treatment anchor	
Efavirenz	39 (86.67%)
Nevirapine	5 (11.11%)
Lopinavir/ritonavir	1 (2.22%)
Days on antiretroviral therapy prior to study entry	84 (17-422)

### Phenotypic and functional changes in unstimulated PBMC

**Figure 1:**
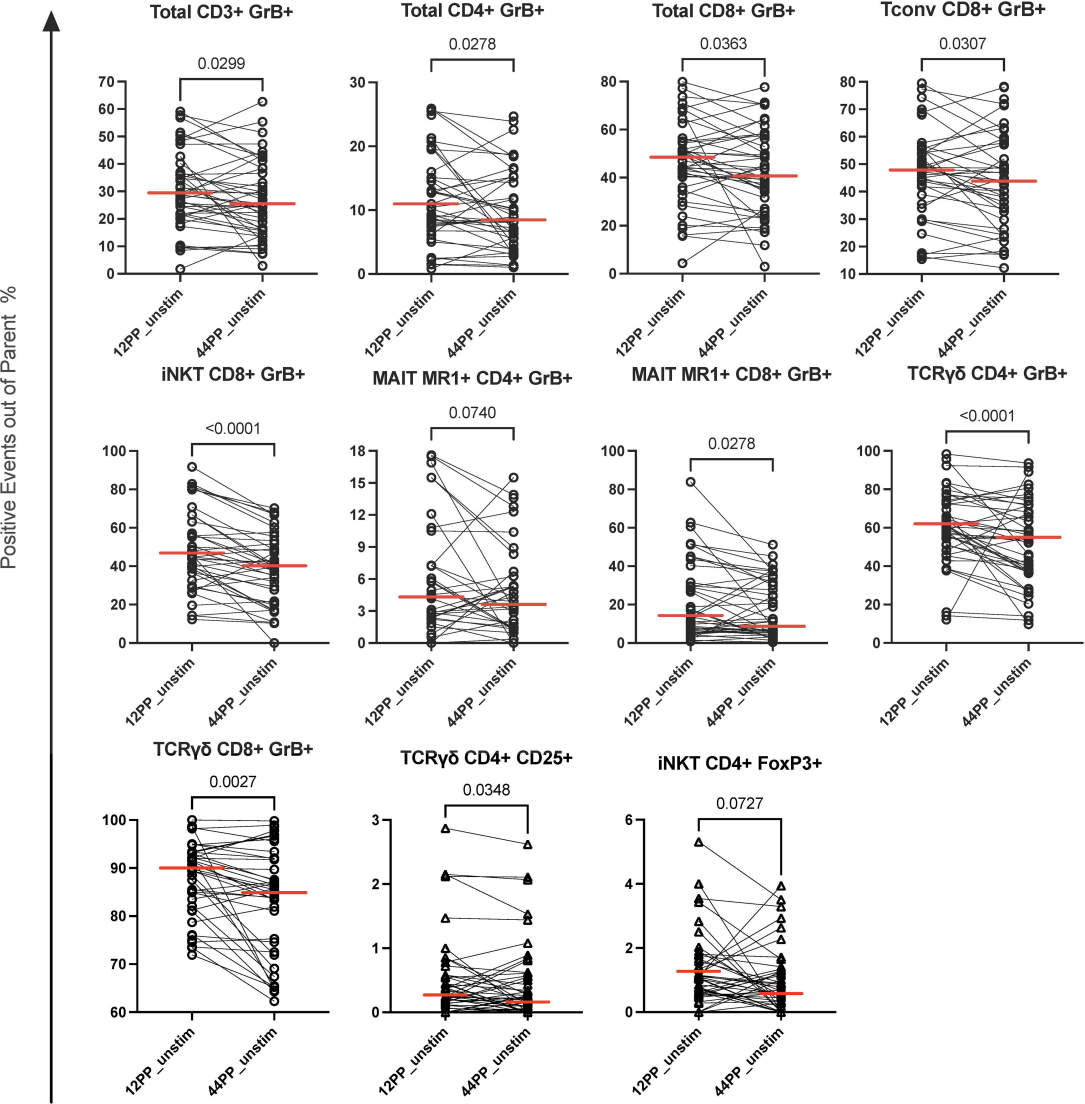
Changes in the frequency of T cell and antigen-presenting cell populations from pre- to post-TPT in unstimulated PBMC of PPWWHIV and LTBI. Data were derived from 45 participants who received TPT between 12 and 40 weeks postpartum. The graphs show pairs of individual data points of the T cell subsets identified in the graph titles. Horizontal red lines indicate medians. FDR p values shown in the panels were calculated with Friedman test for paired observations with nonparametric distribution corrected for multiple comparisons. TPT, TB preventive therapy; PPWWHIV, postpartum women with HIV; LTBI, latent TB infection. 12PP, 12 weeks postpartum; unstim, unstimulated conditions.

We analyzed the frequencies of Th1 (IFNγ+), Th17 (IL17A+), and cytotoxic (Granzyme B+; GrB+) conventional T cells (Tconv), γδ T, iNKT, MR1+ and MR1- MAIT, NKT and NK cells. We also analyzed the expression of FOXP3, CD25, PD1, and IL10 markers of regulation. The most prominent change in the distribution of T cell subsets in PBMC between 12 and 44 weeks postpartum corresponding to pre- and post-TPT administration, respectively, was a significant decrease in the proportions of total CD3+GrB+, CD4+GrB+ and CD8+GrB+ T cells (**Figure 1**). The analysis of T cell subsets identified that CD4+ γδ and MR1+ MAIT cells; and CD8+ Tconv, NKT, iNKT, γδ and MR1+ MAIT cells were responsible for the overall decrease in GrB expression (**Figure 1**). We also noted a decrease in CD4+CD25+ γδ T cells with potential regulatory function (35), and in CD4+FOXP3+ iNKT regulatory T cells (Treg) (36) (**Figure 1**). There were no significant phenotypic changes in NK cells.

Among APC, we analyzed the frequencies of monocytes (classical, intermediate and nonclassical) and dendritic cells (cDC1, cDC2, and pDC) expressing CD80, PDL1, IL1β, IL10, and TNFα. We observed a significant decrease in the proportion of TNFα+ cDC2 from pre- to post-TPT (FDR p = 0.09; not depicted**).**

### Phenotypic and functional changes in TB-stimulated PBMC

Compared to unstimulated PBMC, *in vitro* TB stimulation increased activated and/or regulatory Tconv, unconventional T cells, NK cells and APC, indicating the potential of both adaptive and innate immune cells to contribute to the immune response of TB infection (**Supplementary Figure 2**).

**Figure 2:**
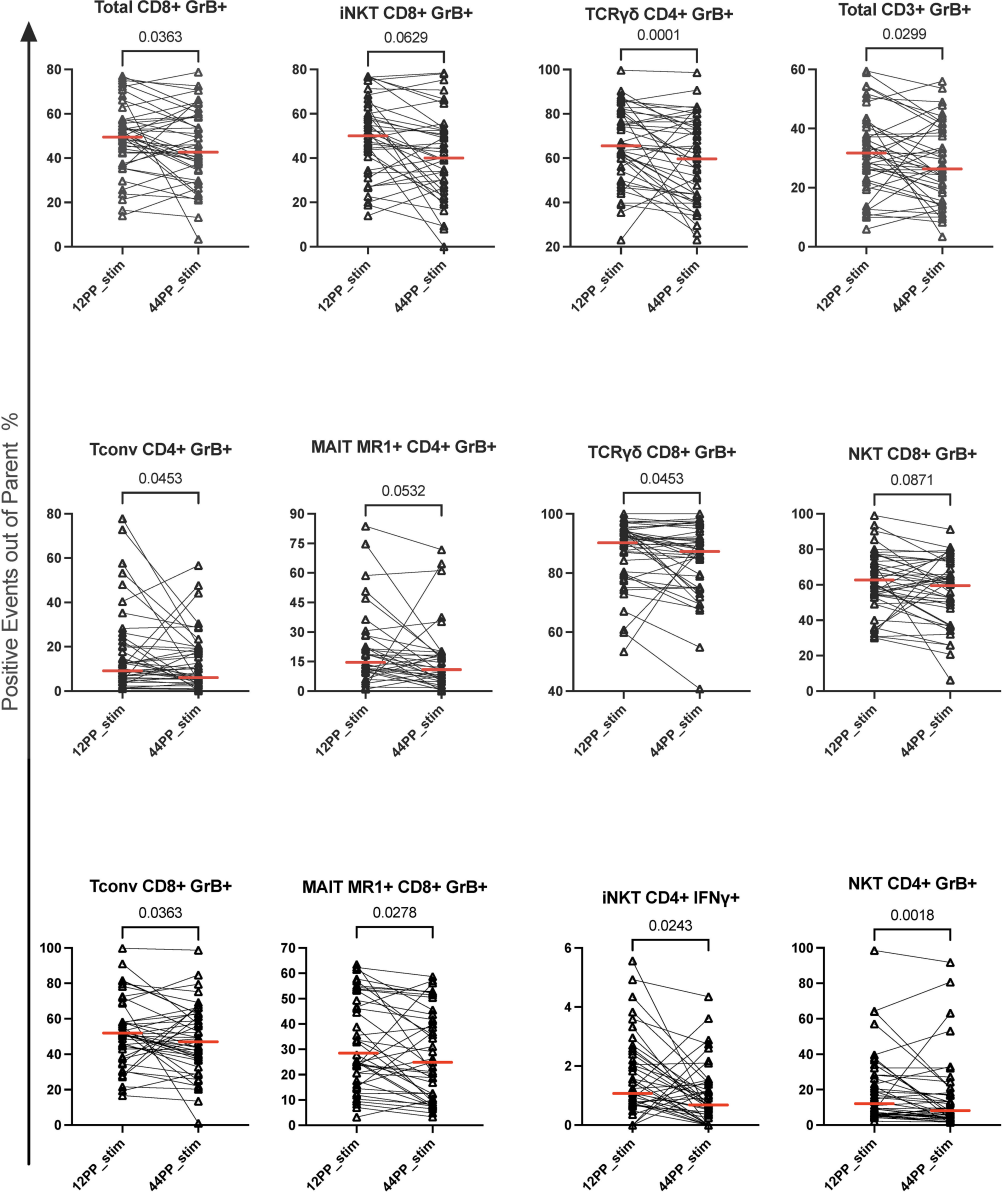
Changes in the proportions of TB-specific T cells from pre- to post-TPT in PPWWHIV and LTBI. Data were derived from 45 participants. The graphs show individual data points of the TB-specific T cell subsets after subtraction of unstimulated from TB-stimulated results. The subsets are identified in the graph titles. The abscissa shows the time points: pre-TPT (12PP) and post-TPT (44PP). Horizontal red lines indicate medians. p values were calculated with Wilcoxon matched-pairs signed rank test. TPT, TB preventive therapy; PPWWHIV, postpartum women with HIV; LTBI, latent TB infection. 12PP, 12 weeks postpartum; 44PP, 44 weeks postpartum; stim, TB-stimulated conditions.

TPT significantly decreased the frequencies of TB-stimulated GrB-expressing total CD3+ and CD8+ T cells (FDR p ≤ 0.04; **Figure 2**) and marginally decreased CD4+GrB+ T cells (FDR p=0.105; not depicted). The analysis of T cell subsets revealed decreased frequencies of GrB-expressing CD4+ and CD8+ Tconv, CD4+ and CD8+ γδ T cells, CD4+ and CD8+ MR1+ MAIT, CD4+ and CD8+ NKT, and CD8+ iNKT cells (**Figure 2**). The proportions of TB-stimulated CD4+IFNγ+ iNKT cells also significantly decreased (**Figure 2**). TB-stimulated CD4+IFNγ+ Tconv and CD4+IFNγ+MR1+ MAIT cells decreased from pre- to post-TPT without quite reaching statistical significance (FDR p of 0.12 and 0.14, respectively; not depicted). Among unconventional T cell subsets with immune regulatory function (37–39), the frequencies of TB-stimulated CD4+PD1+ iNKT, CD4+CD25+ and CD8+CD25+FOXP3+ MAIT MR1+ cells decreased, while CD8+CD25+ NKT cells increased (**Supplementary Figure 3**). The proportions of TB-stimulated TNFα+ cDC2 increased from pre- to post-TPT (FDR p= 0.09; not depicted) but none of the other APC subsets displayed responses to TPT.

**Figure 3:**
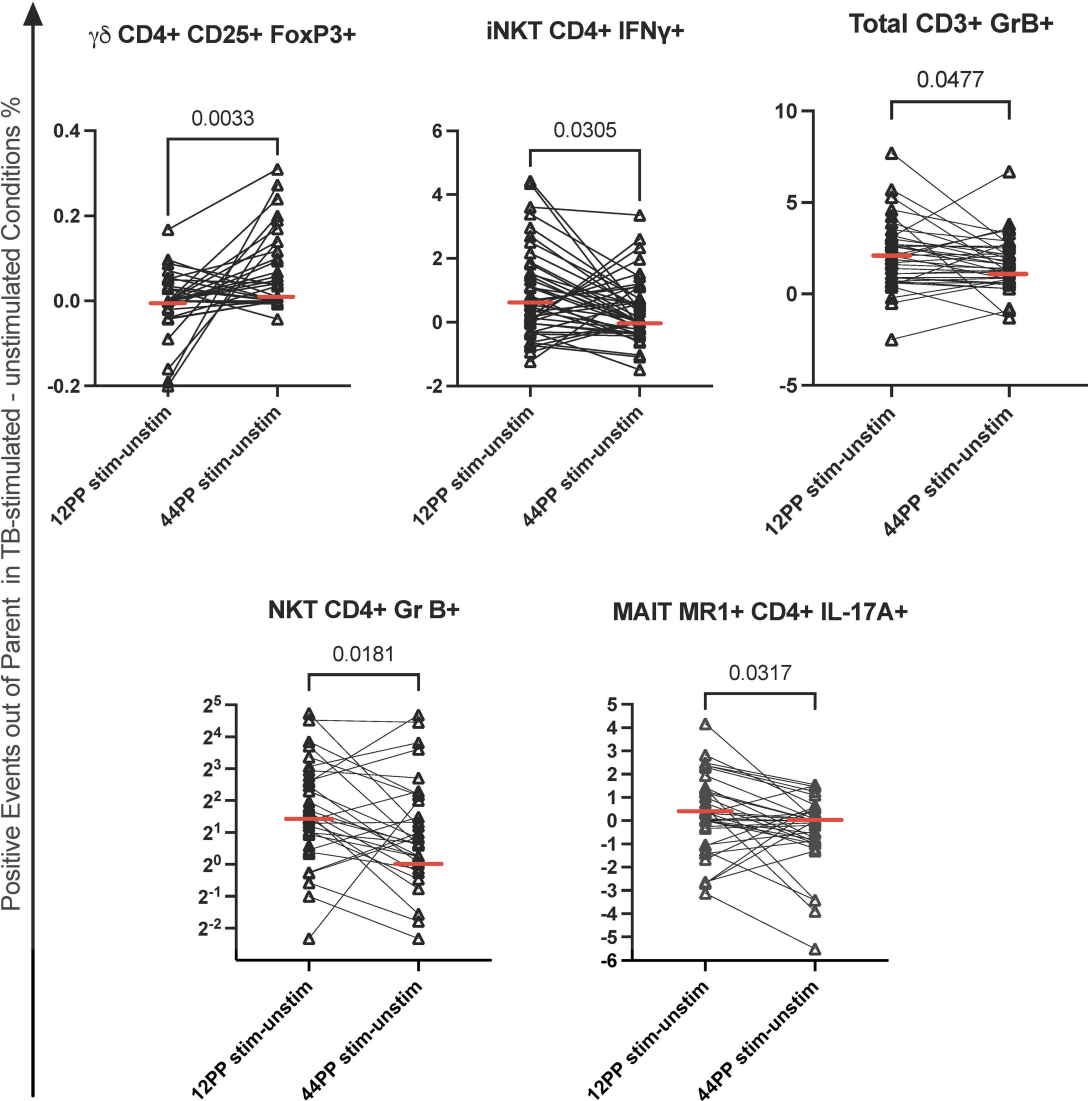
Decrease in the frequencies of TB-stimulated effector T cells from pre- to post-TPT in PPWWHIV and LTBI. Data were derived from 45 participants. The graphs show individual data points of the frequencies of the TB-stimulated T cell subsets identified in the graph titles before TPT (12PP) and after TPT (44PP) as indicated on the abscissa. Horizontal red lines indicate medians. FDR p values were calculated with Friedman test for repeated measures corrected for multiple comparisons. Additional immune cell subsets are shown in **Supplementary Figure 3**. TPT, TB preventive therapy; PPWWHIV, postpartum women with HIV; LTBI, latent TB infection. 12PP, 12 weeks postpartum; 44PP, 44 weeks postpartum; stim, TB-stimulated conditions.

To complement and confirm the results above, we performed a supplementary analysis of TB-specific responses by subtracting the frequency of activated cells in unstimulated PBMC from frequencies in TB-stimulated PBMC. We observed, significant decreases in the frequencies of total CD3+GrB+ T cells, CD4+GrB+ NKT, CD4+IFNγ+ iNKT, CD4+IL17A+ MR1+ MAIT from pre- to post-TPT and increases in the frequencies CD4+CD25+FOXP3+ γδ T cells (**Figure 3**). These results confirmed the decrease in TB-specific GrB+ and IFNγ+ unconventional T cells and increase in TB-specific innate Treg (35) and uncovered a decrease in TB-specific IL17A+ innate T cells.

To rule out bystander activation in the TB-stimulated immune cell subsets that showed significant changes from week 12 to week 44 postpartum, we performed correlation analyses of each of the subsets with TB-stimulated CD4+IFNγ+ Tconv, representing the largest TB-specific T cell population and with the highest capacity to engage other cells in the immune response through cytokine secretion. The analyses did not show any significant positive associations (not depicted).

### Effect of TPT on PBMC cytokine secretion

**Figure 4:**
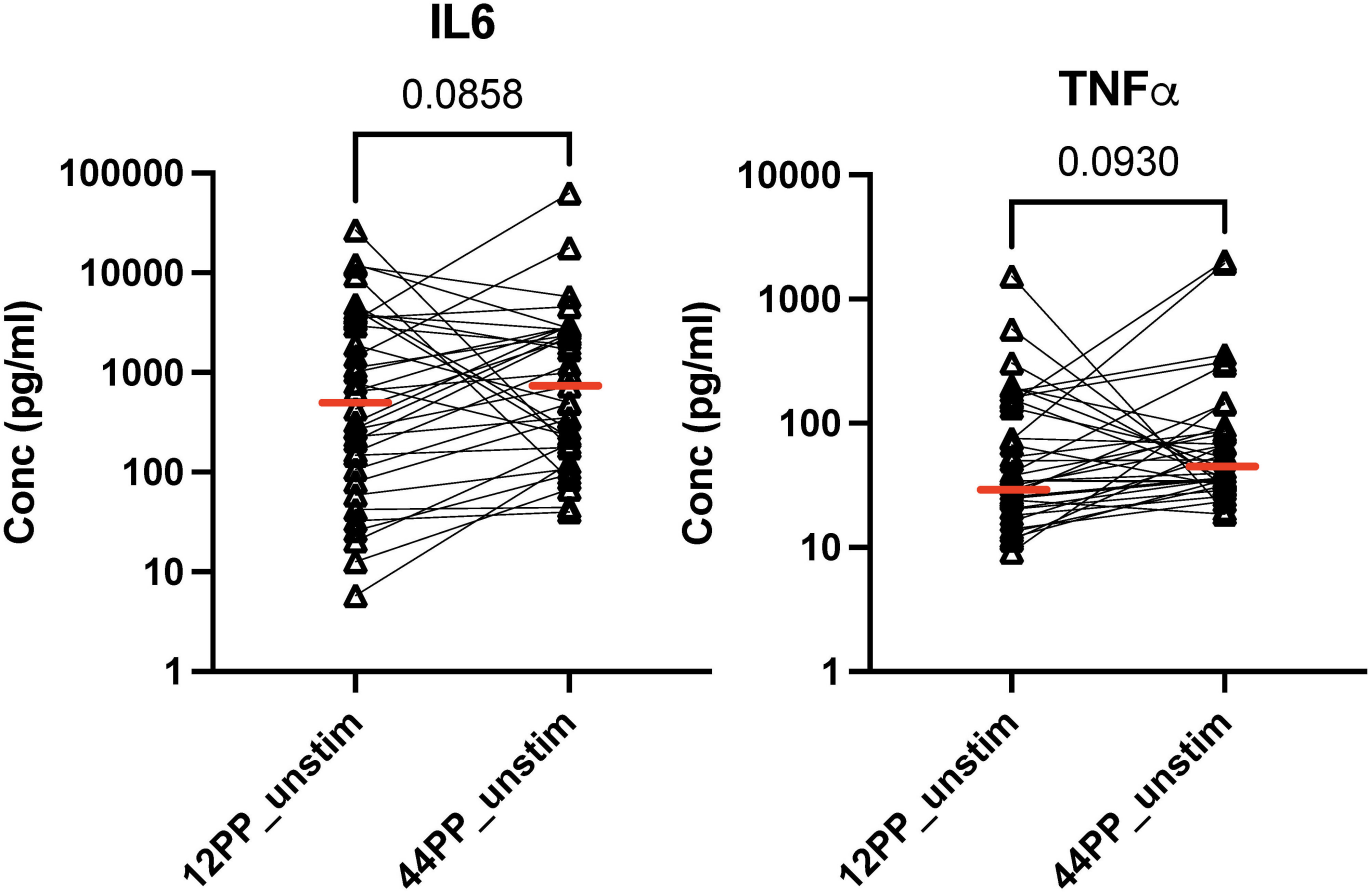
Cytokines secreted in culture supernatants of unstimulated PBMC pre- and post-TPT in PWWHIV and LTBI. Data were derived from 45 participants. The graphs show individual data points of cytokine concentrations identified in the graph titles before TPT (12PP) and after TPT (44PP) in unstimulated (unstim) and TB-stimulated (stim) as indicated on the abscissa. Horizontal red lines indicate medians. FDR p values were calculated with Friedman test for repeated measures corrected for multiple comparisons. TPT, TB preventive therapy; PWWHIV, postpartum women with HIV; LTBI, latent TB infection. 12PP, 12 weeks postpartum; 44PP, 44 weeks postpartum; stim, TB-stimulated conditions.

We analyzed TB-stimulated and unstimulated PBMC secretion of nine cytokines and two checkpoint inhibitors, including CTLA4, GMCSF, IL2, IL4, IL6, IL10, IL17, IFNγ, PD1, TGFβ, and TNFα. TNFα and IL6 secretion in unstimulated PBMC increased between 12 and 44 weeks postpartum (**Figure 4**). There were no significant changes in the secretion of other markers in TB-stimulated or unstimulated PBMC.

## Discussion

**Figure 5:**
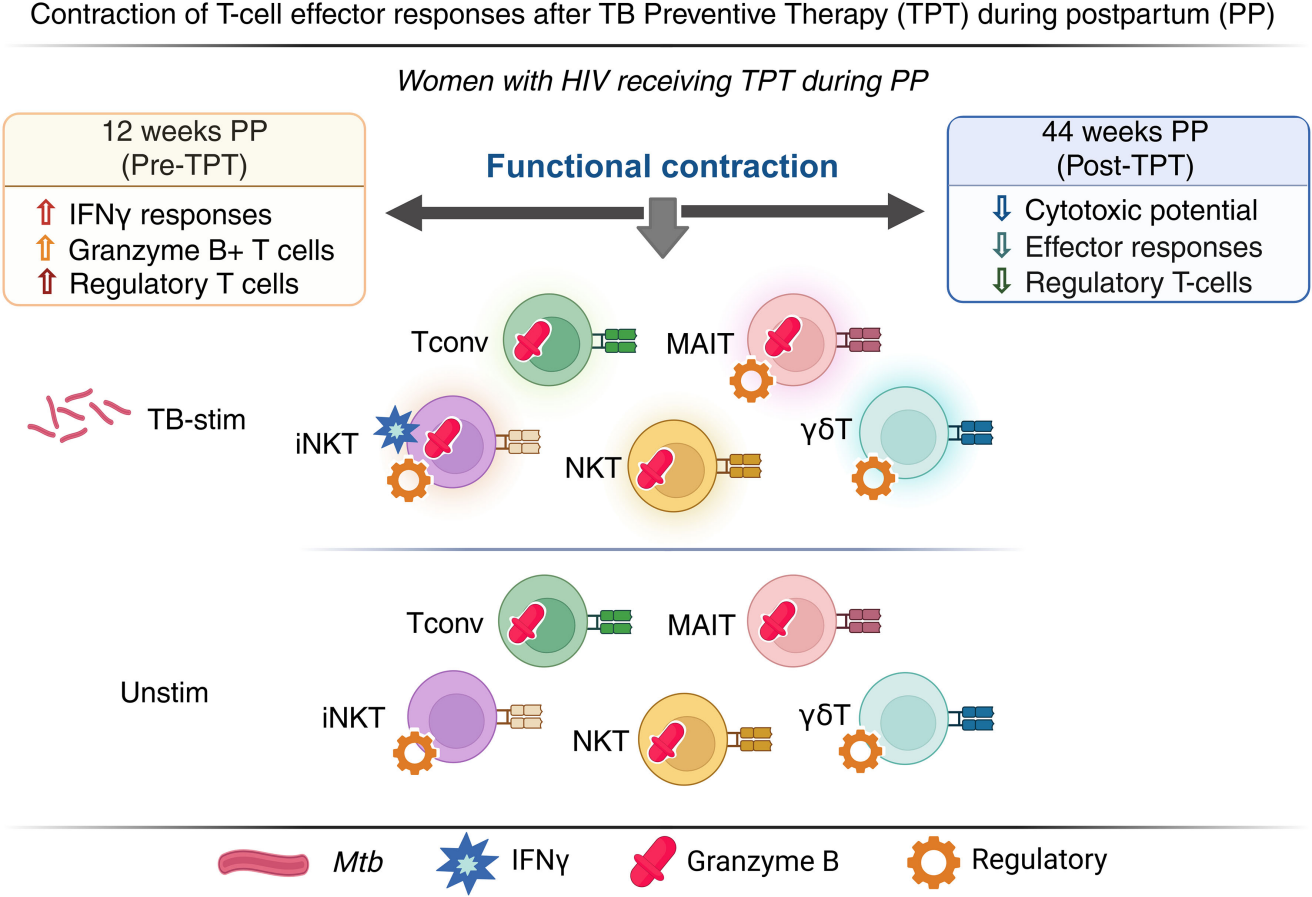
Summary diagram of the effect of TPT on TB-specific and nonspecific immune responses.

TPT decreased Th1 responses of Tconv and unconventional T cells, including IFNγ production by iNKT and cytotoxicity effected by γδ, iNKT, NKT and MAIT cells in PPWWHIV with LTBI. TPT was also associated with a decrease in TB-specific Treg suggesting an overall contraction of the TB-specific memory compartment (**Figure 5**). We found that unconventional T cell immunologic memory may play a role in protection against progression of TB infection in this group. In addition to Tconv, unconventional T cell responses to TPT were independent of CD4+ Tconv responses, which ruled out the potential of bystander activation. This observation suggests additional T cell subsets that TB vaccine may target for increased protection against TB.

The decrease in TB-specific IFNγ responses induced by TPT or treatment of active TB was previously assumed to represent a decrease in Tconv responses (14, 15, 40–42). We found that iNKT cells lost TB-specific IFNγ responses in higher proportions than Tconv and that MR1+ MAIT cells also contributed to the decrease in TB-specific IFNγ production after TPT in PPWWHIV. The corollary of these observations is that innate immune T cells may require sustained antigenic exposure *in vivo* as much or more than Tconv to maintain TB-specific effector responses in PPWWHIV. This finding may be particularly relevant to PWHIV, because their TB-specific CD4+ Tconv are deemed to be specifically targeted for destruction by the virus (8, 43) which may amplify the importance of unconventional T cells in protection against TB reinfection in this group.

We found a generalized decrease in GrB-expressing T cells both in TB-stimulated and unstimulated PBMC from pre- to post-TPT. GrB has a dual role in protection against TB. Cytotoxic T cells use GrB to lyse bacterially infected cells. Moreover, GrB disrupts the metabolism and protein synthesis of *M. tuberculosis* leading to bacterial death (44, 45). This mechanism of protection seems to stabilize the granulomas (46) and is particularly active in LTBI (47). We postulated that increases in the frequency of TB-specific GrB+ cells in the context of LTBI were large enough to translate into an overall increase in GrB+ T cells in PBMC. Thus, when the number of TB-specific GrB+ T cells decreased in response to TPT, the reduction was reflected in the relative frequency of GrB+ T cells in circulating PBMC. This finding is supported by a recent study showing that acute phase inflammatory markers decrease from pre- to post-TPT in plasma of PWHIV and LTBI (48). It is important to note that other elements common to our study participants, HIV infection and transition from antepartum to postpartum, could not explain the decrease in GrB expression. In contrast, both transition of antepartum to postpartum (49) and control of HIV infection (50) have been associated with increased cytotoxic activity and GrB expression Collectively, these observations attest to the significant effect of LTBI not just on TB-specific but on overall host immune responses.

We did not observe significant changes in the TB-specific IL17A secretion in culture supernatants after TPT, which was in agreement with previous reports (15). However, the analysis of the IL17A responses to TB stimulation in unconventional T cells, revealed a decrease in the frequency of TB-specific MR1+CD4+IL17A+ MAIT cells from pre- to post-TPT, underscoring the potential role of these cells in protection against TB.

A previous study showed that TPT did not change production of the IL10 regulatory cytokine in IGRA supernatants (15), which we also found to be true in our participants both for IL10 and for the TGFβ regulatory cytokines. However, using a more granular flow cytometric approach, we found variability in the response of TB-specific innate Treg from pre- to post-TPT. Unconventional Treg have only recently been described both in tumors (35, 37, 39), and in response to *M. tuberculosis* stimulation (38). We identified post-TPT decreases in the abundance of iNKT cells expressing PD1 previously shown to have Treg activity in tumors (39) and MR1+ MAIT cells expressing CD25 and FOXP3 markers previously shown to have Treg activity when stimulated by *M. tuberculosis* (38). In contrast, TB-stimulated γδT cells co-expressing CD25 and FOXP3 previously shown to have Treg activity (35) and CD25+ NKT cells with Treg activity (37) increased after TPT. The decrease in TB-specific Treg was in agreement with the general decline in TB-specific T cells post-TPT. Conversely, the increase of TB-specific innate Treg might reflect the need to maintain control of the previously described bystander activation and/or cross-reactive responses of TB-specific memory T cells (51). Notably, we did not find changes in conventional Treg, suggesting the short-term resilience to TPT of this cell subset.

In addition to TPT, the cumulative time on ART and recovery from pregnancy-induced immunologic changes may have contributed to the contraction of the Treg compartment, which generally expands during uncontrolled HIV replication and pregnancy (52, 53). The increase in IL6 and TNFα in culture supernatants of unstimulated PBMC may also reflect a delayed correction of the immune-suppressive effect of pregnancy.

This study has strengths and limitations. It includes the first comprehensive analysis of immunologic changes generated by TPT in PPWWHIV and LTBI, which provided a large dataset of innate and adaptive immune responses in a relatively large number of PPWWHIV. However, the follow up was limited to the period of TPT. Another limitation was the fact that all participants were from Sub-Saharan Africa, such that our findings may not apply to people of different races or ethnicities. Although we did not have information on HIV plasma RNA after study entry, treatment compliance monitored at monthly visits by self-report indicated >90% adherence suggesting that HIV replication remained under effective control throughout the study. Moreover, CD4+ T cells increased, which was consistent with sustained control of the viral replication.

In conclusion, we determined that TPT was associated with a decrease in TB-specific immune responses in PPWWHIV. This finding suggests that these individuals may benefit from boosting their immune responses through vaccination. We also found that TB-specific unconventional T cell immunologic memory accounted for most of the changes in TB-specific cell-mediated immunity immediately after completion of TPT, suggesting its important role in the control of TB infection. Additional studies are needed to establish if this finding applies to people infected with TB in the absence of HIV infection, because it may expand the parameters used to assess the immunogenicity of TB candidate vaccines to include TB-specific unconventional T cells.

## Data Availability

The raw data supporting the conclusions of this article will be made available by the authors, without undue reservation.
