# Supplementary material for: Tuberculosis preventive therapy in postpartum women with HIV modifies M. tuberculosis-specific and nonspecific immune responses
Source: Front Immunol. 2026 Mar 11;17:1722621. doi: 10.3389/fimmu.2026.1722621 (PMC13012941; doi:10.3389/fimmu.2026.1722621)
Supplement: Supplementary file 1 [file DataSheet1.docx]

**Supplemental Table 1. Markers used for phenotypic and functional characterization of the immune subsets.**

| **Cell Subset** | | **Lineage** | **Function** |
| --- | --- | --- | --- |
| T cells | Tconv | CD3+ TCR*γδ-* TCR Vα7.2- CD161-TCRVα24Jα18- | CD8, IFNγ, IL17, Granzyme B, CD25, FOXP3, IL10, PD1 |
|  | *γδT* | CD3+TCR*γδ*+ |  |
|  | *MAIT* | CD3+TCR*γδ-* TCR Vα7.2+MR1-tetramer+/- |  |
|  | NKT | CD3+ TCR*γδ-* TCRVα24Jα18-CD161+ |  |
|  | *iNKT* | CD3+ TCR*γδ-* Vα24-Ja18+ |  |
| NK | | Lin-CD14-CD16+ and/or CD56+ | IL1β, PDL1, IL10, TNFα, IFNγ,  Granzyme B |
| APC | *Monocyte* | Lin-HLADR+CD14+ |  |
|  | *cDC1* | Lin-HLADR+CD14-CD123-CD141+ |  |
|  | *cDC2* | Lin-HLADR+CD14-CD123-CD1c+ |  |
|  | *pDC* | Lin-HLADR+CD14-CD123+ |  |

**Supplemental Table 2. Monoclonal antibodies used in flow cytometry panels.**

| **Sl. No.** | **Antibody** | **Cat. No.** | **Company** |
| --- | --- | --- | --- |
| 1 | Anti-CD3-Ax700 | 557943 | BD Biosciences |
| 2 | Anti-CD8-BV570 | 301038 | BioLegend |
| 3 | Anti-TCRγδ-BV711 | 745505 | BD Biosciences |
| 4 | Anti-TCRVα7.2-PE-CD594 | 351730 | BioLegend |
| 5 | Anti-CD161-PE-Cy5 | 306710 | BioLegend |
| 6 | Anti-TCRVα24Jα18-PE-Cy7 | 342914 | BioLegend |
| 7 | Anti-CD25-BV786 | 356140 | BioLegend |
| 8 | MR1 tetramer-PE | TS-HMRV2-1 | NIH Tetramer Core Facility, Emory University |
| 9 | Anti-PD1-BV650 | 564324 | BD Biosciences |
| 10 | Anti-HLA-DR-BV650 | 307650 | BioLegend |
| 11 | Anti-CD14-BV785 | 367142 | BioLegend |
| 12 | Anti-CD16-FITC | 302006 | BioLegend |
| 13 | Anti-CD56-PerCP-Cy5.5 | 318322 | BioLegend |
| 14 | Anti-CD123-BV605 | 306026 | BioLegend |
| 15 | Anti-CD141-APC | 344106 | BioLegend |
| 16 | Anti-CD1c-PE-Cy7 | 331516 | BioLegend |
| 17 | Anti-CD19-Ax700 | 363033 | BioLegend |
| 18 | Anti-CD20-Ax700 | 302322 | BioLegend |
| 19 | Anti-IL10-BV421 | 501422 | BioLegend |
| 20 | Anti-IFNγ-BV605 | 506542 | BioLegend |
| 21 | Anti-granzyme B-FITC | 372206 | BioLegend |
| 22 | Anti-FoxP3-APC | 17-4777-41 | Thermo Fisher Scientific |
| 23 | Anti-IL17-APC Cy7 | 512320 | BioLegend |
| 24 | Anti-IFNγ-BV570 | 502534 | BioLegend |
| 25 | Anti-IL1β-PE | 567779 | BD Biosciences |
| 26 | Anti-granzyme B-PE-CF594 | 372216 | BioLegend |
| 27 | Anti-TNFα-APC-Cy7 | 502944 | BioLegend |


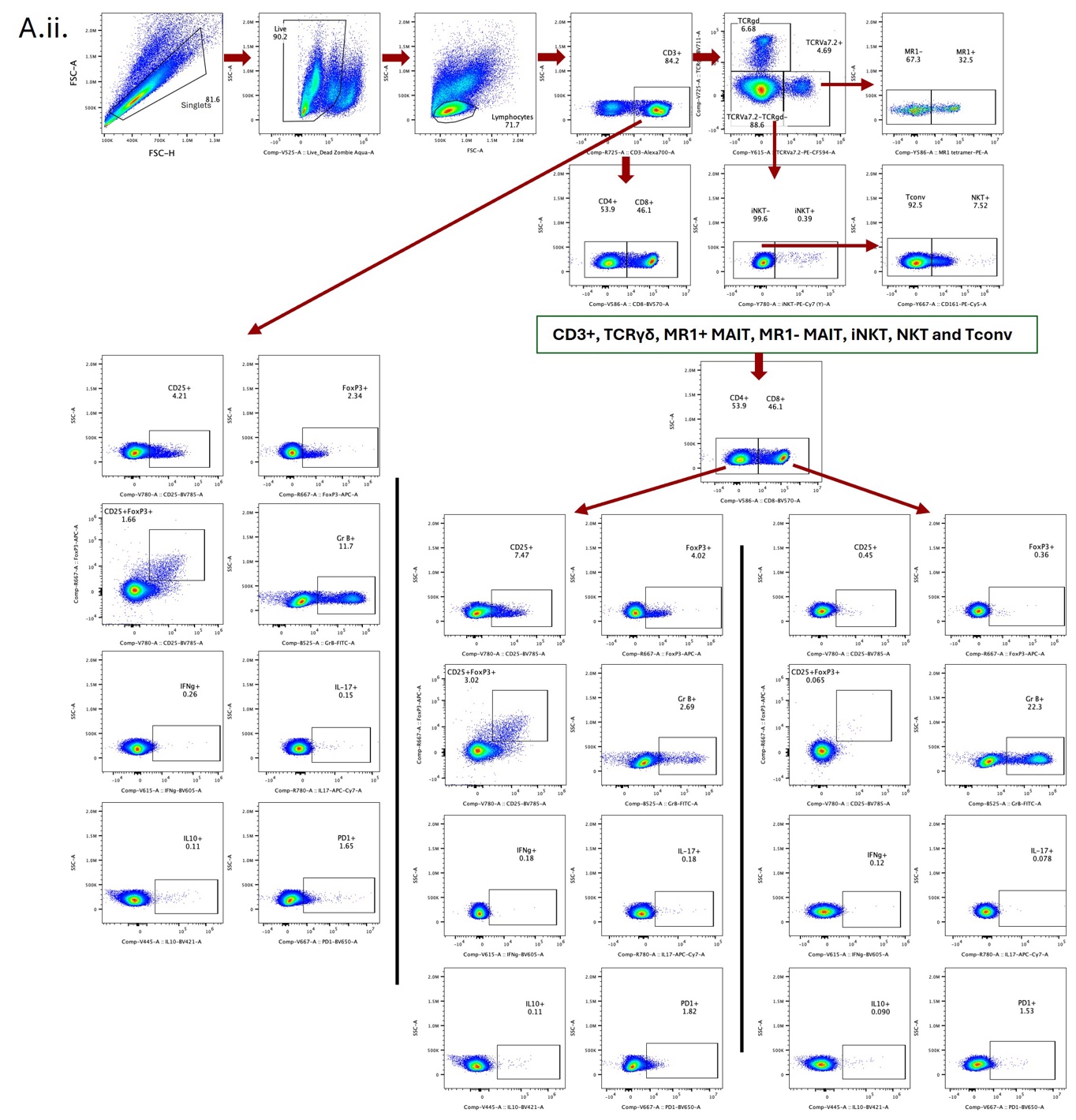


**A**


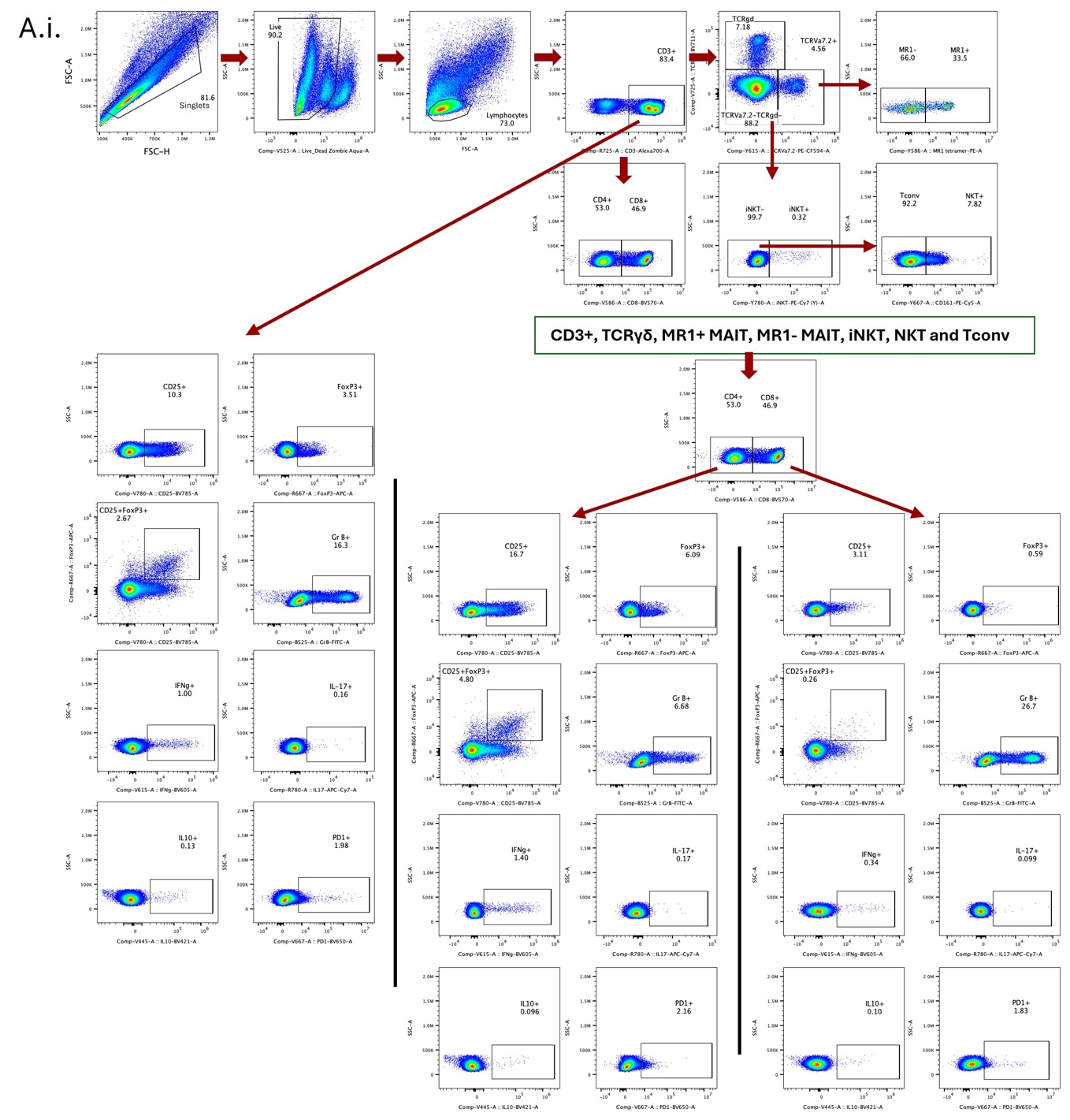

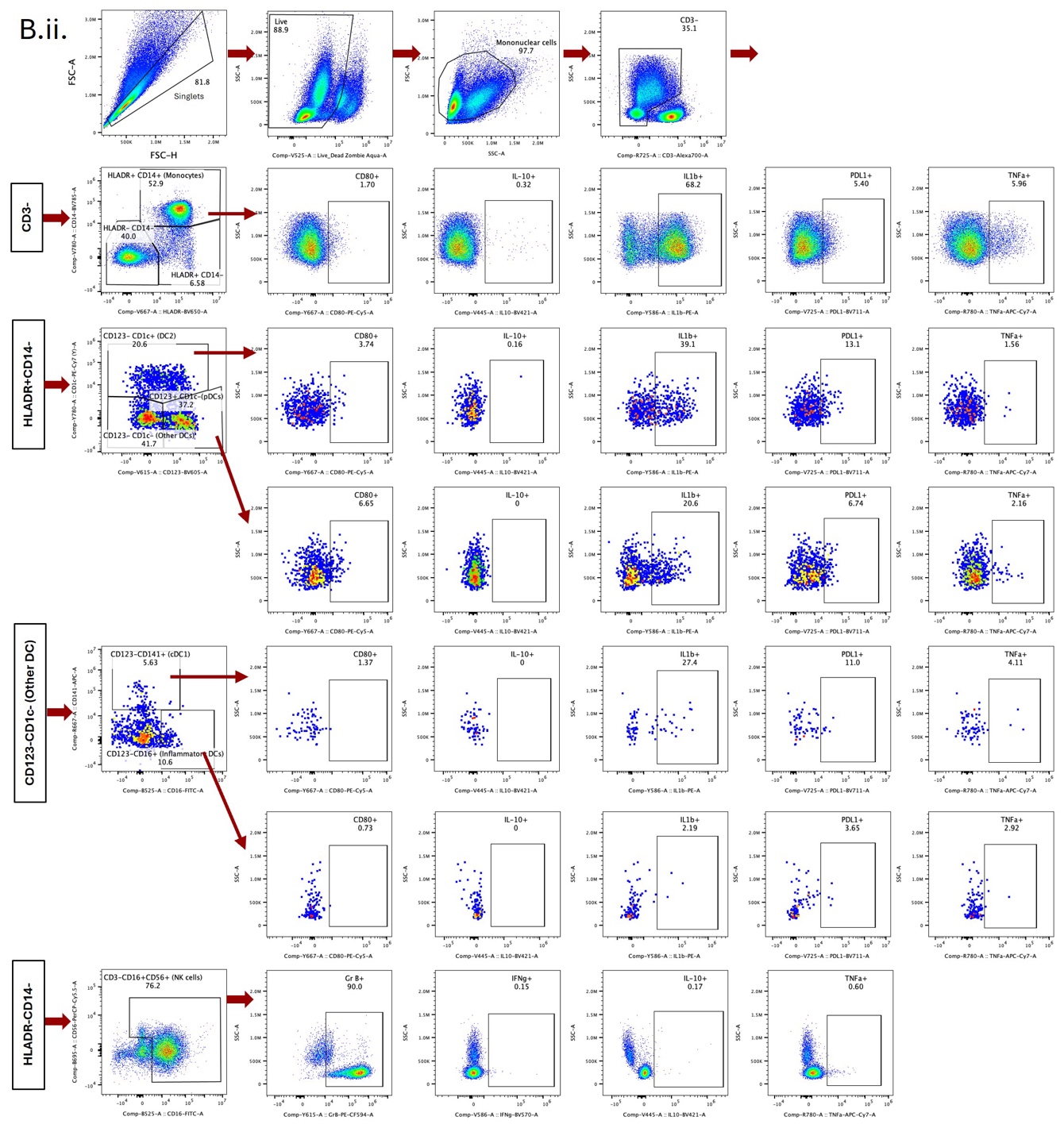


**B**

**A**

**C**


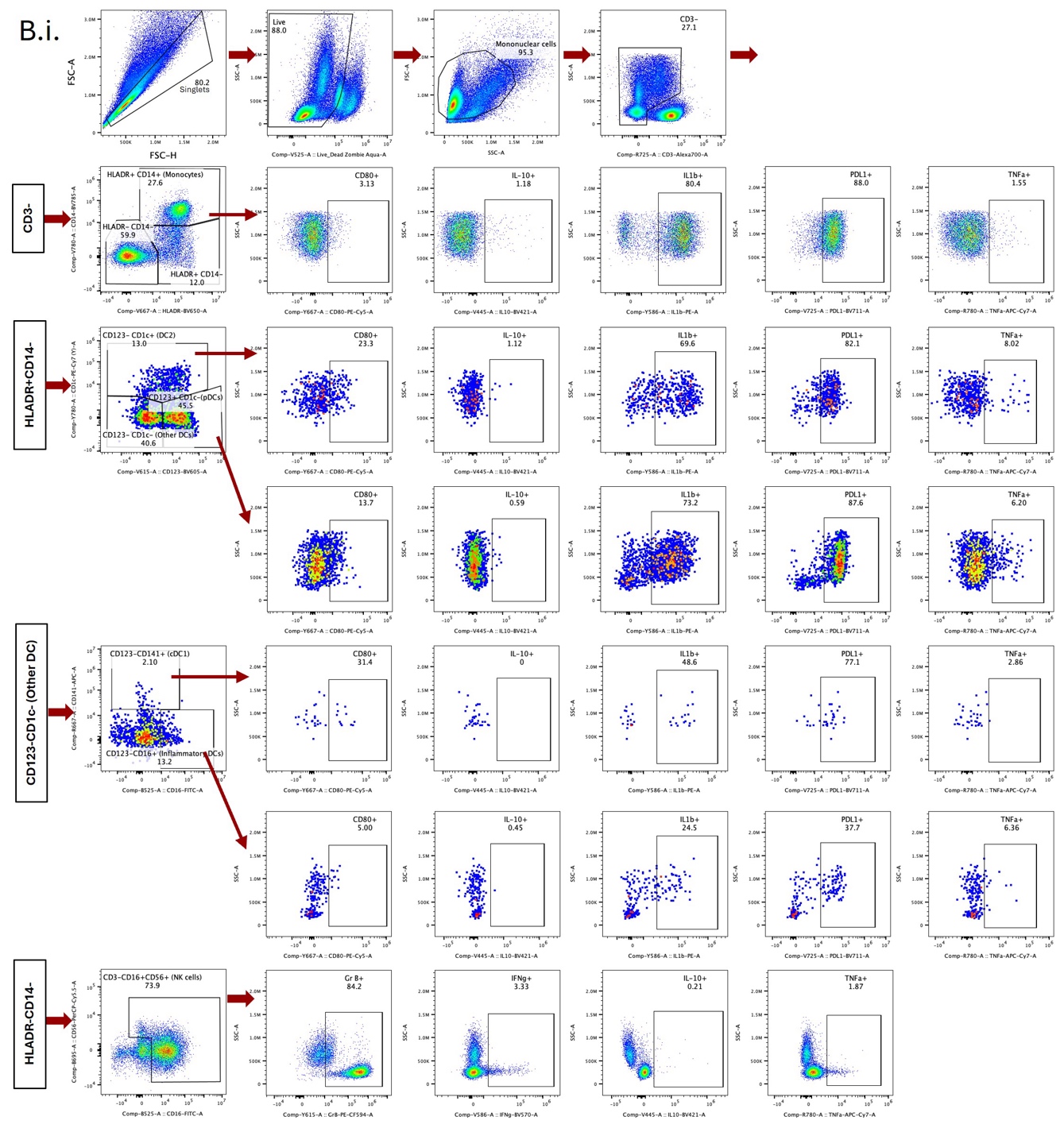


**D**

**Supplemental Figure 1. Gating strategies.**

**Panel** **A** shows T cell gating in unstimulated conditions.

**Panel** **B** shows T cell gating in TB-stimulated conditions.

**Panel C** shows APC gating in unstimulated conditions.

**Panel D** shows APC gating in TB-stimulated conditions.

**Supplemental Figure 2. Innate and adaptive immune cell responses to in vitro TB antigenic stimulation in PWWHIV and LTBI before TPT initiation.** Data were derived from 45 participants. The graphs show individual data points of the frequencies of the cell subsets identified in the graph titles in unstimulated and TB-stimulated conditions as indicated on the abscissa. Horizontal red lines indicate medians. FDR p values were calculated with Friedman test for repeated measures corrected for multiple comparisons. Other immune cell subsets are shown in **Figure 2**. Abbreviations: TPT=TB preventive therapy; PWWHIV=postpartum women with HIV; LTBI=latent TB infection. 12PP=12 weeks postpartum; unstim=unstimulated conditions; stim=TB-stimulated condition.

**Supplemental Figure 3. Changes in the frequencies of TB-activated regulatory T cells from pre- to post-TPT in PPWWHIV and LTBI.** Data were derived from 45 participants. The graphs show individual data points of the frequencies of the TB-stimulated T cell subsets identified in the graph titles before TPT (12PP) and after TPT (44PP) as indicated on the abscissa. Horizontal red lines indicate medians. FDR p values were calculated with Friedman test for repeated measures corrected for multiple comparisons. Other immune cell subsets are shown in **Figure 2**. Abbreviations: TPT=TB preventive therapy; PPWWHIV=postpartum women with HIV; LTBI=latent TB infection. 12PP=12 weeks postpartum; 44PP=44 weeks postpartum; stim=TB-stimulated conditions.
